# Double dystonia secondary to risperidone: acute laryngeal dystonia and oculogyric crisis.

**DOI:** 10.1192/j.eurpsy.2023.2263

**Published:** 2023-07-19

**Authors:** L. Tardon, O. Marco, L. Navarro, T. Fernandez, O. de Juan, M. Bioque, H. Andreu

**Affiliations:** Hospital Clínic de Barcelona, Barcelona, Spain

## Abstract

**Introduction:**

Acute laryngeal dystonia due to antipsychotics is an uncommon but potentially lethal form of extrapyramidal reaction. The initial symptoms may be subtle but progressively appear difficulties in phonation, stridor and dyspnea which are often life-threatening.

**Objectives:**

To describe a case of acute laryngeal dystonia and oculogyric crisis secondary to risperidone.

**Methods:**

The present study is a case report of a patient admitted for schizophrenia who was presented a laryngeal dystonia and oculogyric crisis after being treated with 5mg risperidone. We also searched previously case reports, series and systematic reviews of laryngeal dystonia using a pubmed query.

**Results:**

A 30-year-old Caucasian woman who was admitted for schizophrenia presented rhinolalia, oropharynx paresthesias, mild dyspnea without stridor, and prolonged involuntary upword desviation of the eyes. All these symptoms started within 24 hours of starting risperidone 5mg per day. A laryngoscopy showed abnormal motion of the vocal cords that suggested laryngeal dystonia. Symptoms remitted after administration of intramuscular biperiden 4mg. Risperidone was later switched to olanzapine because of better psychomotor side-effect profile.

**Conclusions:**

Laryngeal dystonia is a medical emergency requiring early diagnosis and immediate treatment. Anticholinergic agents should be carried out, without waiting for the results of complementary tests. The route of administration can be intramuscular or intravenous. This complication should be always kept into account when a patient is taking any antipsychotic, and remembered for the antipsychotic election in following treatments.

**Disclosure of Interest:**

None Declared

